# Heart regeneration, stem cells, and cytokines

**DOI:** 10.1186/2050-490X-2-6

**Published:** 2014-04-02

**Authors:** Na Li, Chuan Wang, LiXin Jia, Jie Du

**Affiliations:** Capital Medical University Affiliated Beijing Anzhen Hospital, Anzhenli, Chaoyang District, Beijing, 100029 China; Lung and Vessel Diseases, Beijing Research Institute of Heart, Beijing, 100029 China; The Key Laboratory of Remodeling-related Cardiovascular Diseases, Capital Medical University, Ministry of Education, Beijing, China

**Keywords:** Cytokines, Heart regeneration, Stem cells, Paracrine mechanisms

## Abstract

The human heart has limited regenerative capacity, which makes the reparative response after the cardiac infarction quite challenging. During the last decade, stem cells have become promising candidates for heart repair, owing to their potent differentiation capacity and paracrine cytokine secretion. Among the different types of stem cells, mesenchymal stem cells have high proliferative potential and secrete numerous cytokines, growth factors, and microRNAs. The paracrine cytokines play important roles in cardiac regeneration, neovascularization, anti-apoptosis, and anti-remodeling mechanisms, among others. This review summarizes the cytokines secreted by stem cells and their relative signaling pathways, which represent key mechanisms for heart regeneration and may serve as a promising future therapeutic strategy for myocardial infarction patients.

## Review

### Introduction

Myocardial infarction (MI) causes the loss of cardiac tissue and scar formation, which ultimately lead to heart failure. According to the World Health Organization, heart failure initiated by MI and coronary artery disease accounts for 29% of deaths worldwide [1]. However, human heart tissue does not regenerate spontaneously, thus “regenerative medicine” represents a promising alternative treatment for MI [2]. Cardiac tissue regenerative medicine involves cardiomyocyte regeneration, neovascularization, and paracrine cytokines, which have anti-inflammatory, anti-apoptotic, and anti-remodeling effects [3]. During the last decade, stem cells have become promising candidates for regenerative medicine not only because of their capacity of differentiation toward cardiomyocyte and vascular cell lineages but also their capacity for releasing such paracrine factors and their anti-arrhythmic effects [4, 5]. Paracrine cytokines and chemokines play pivotal roles in stem cell-related cardiac repair mechanisms.

Although great advances have been made in the identification of novel strategies to save the myocardium and improve the mortality of MI patients, further understanding of the impact of cytokines on myoblast differentiation and the related signaling pathways may provide unique opportunities for reducing cardiac impairment.

The current review summarizes the stem cell-related cytokines and related reparative pathways that represent potential therapeutic targets for cardiac regeneration after MI.

### Cellular sources of cardiac regeneration

#### Cardiac progenitor cells

Shortly after birth, human cardiomyocytes cease proliferating and exhibit a very limited regenerative capacity. However, this concept has been challenged recently. Bergmann et al. used C_14_ to carbon date the DNA of dividing cardiomyocytes. They demonstrated that the diploid cardiomyocyte nuclei were younger than the human subjects, providing good evidence for cardiomyocyte division in adult humans. Mathematical statistics suggested that approximately 1% of cardiomyocytes were renewed per year at age 20 years, and 0.4% at age 75 years [6]. Based on these kinetics, about 45% of cardiomyocytes would be predicted to be renewed over the normal human lifespan, whereas 55% would be cells persisting since birth. In the female heart, myocyte turnover occurs at a rate of 10%, 14%, and 40% per year at 20, 60, and 100 years of age, respectively [7]. After this demonstration of the renewal capacity in adult heart tissue, the existence of cardiac progenitor cells (CPCs) in postnatal hearts has been reported by different groups [8]. CPCs have been identified by surface markers such as such as c-KIT, Isl1 cells, or SCA-1 and their physiological properties such as the ability to efflux fluorescent dye (i.e., side population) [9]. CPCs expressing the tyrosine kinase receptor c-KIT are the most extensively investigated subtype [10]. Beltrami et al. has first identified the c-KIT–positive CSCs in the heart capable of dividing symmetrically and asymmetrically in vitro and differentiating into myocytes, vascular smooth muscle cells (SMCs), and endothelial cells (ECs) [11]. Newly differentiated cardiomyocytes possess the mechanical and electric properties of functionally cardiomyocytes, which improve cardiac function after MI [12]. Some studies indicate that, when transplanted, c-KIT^+^ cells induce large-scale regeneration of myocardial infarcts and contribute to the formation of new myocardium and vessels, whereas others suggest smaller-scale regeneration [10]. Hatzistergos and colleagues demonstrated that MSCs may stimulate endogenous CSCs, including c-KIT CSCs and GATA-4 CSCs, to differentiate into enriched populations of adult cardioblasts that express Nkx2–5 and troponin I both in vivo and in vitro [13]. However, clinical applications of CSCs are limited because of their small number and low proliferation capacity. Nevertheless, CSCs play a pivotal role in the maintenance of cardiac homeostasis and repair.

#### Mesenchymal stem cells

Since the first demonstration that bone marrow-derived mesenchymal stem cells (MSCs) can generate functional cardiomyocytes [14], these cells have become a promising therapeutic candidate for heart disease. In the past decade, many clinical trials have used MSCs to treat MI and heart failure. The results have demonstrated clinical safety and efficacy, but the cardiac function improvements have been limited, ranging from 3% to 15% [15]. Currently, differentiation of MSCs into functional cardiomyocytes remains controversial and the rate of myocardial regeneration appears too minor to explain the significant cardiac function recovery observed after MSC transplantation following MI. Accumulating evidence suggests that the MSC cytokine profile exerts beneficial effects on the prevention of apoptosis and fibrosis as well as improvement of cardiac function [16–18]. For example, Duran et al. recently demonstrated that bone marrow-derived stem cells improve survival, cardiac function, and attenuate remodeling through the secretion of pro-angiogenic factors that stimulate endogenous neovascularization [5].

#### Induced pluripotent stem cells

Fifteen years ago, Murry et al. first demonstrated that fibroblasts could be transdifferentiated into skeletal muscle in vitro by overexpressing the myogenic transcription factor, MyoD [19]. Subsequently, Yamanaka and colleagues proved that pluripotent stem cells could be induced from mouse embryonic or adult fibroblasts by introducing four defined factors, Oct3/4, Sox2, c-Myc, and Klf4, under embryonic stem cell culture conditions [20]. While this groundbreaking finding has opened an exciting research area, its clinical applications remain limited given the transgenic integration and alteration of the endogenous genomic organization. Induced pluripotent stem cells (iPS) can differentiate into cardiomyocytes via mechanisms involving the aforementioned cytokines. The production of the iPS by non-viral methods or a combination of the cytokines may serve as alternatives for iPS cell therapy.

Under hypoxic conditions, stem cells can release growth factors and cytokines such as transforming growth factor (TGF)-β, interleukin (IL)-6, vascular endothelial growth factor (VEGF), fibroblast growth factor (FGF)-2, hepatocyte growth factor (HGF), insulin-like growth factor (IGF), angiopoietin (Ang)-1, stromal cell-derived factor (SDF)-1, matrix metalloproteinase (MMP)-9, and tumor necrosis factor (TNF)-α, among others. These secreted cytokines exert important anti-apoptosis, anti-inflammatory, and anti-remodeling effects in a paracrine manner [21].

### Cytokines, stem cells, and cardiomyocyte regeneration

Cytokines are small cell-secreted molecules that play pivotal roles in cell proliferation, differentiation, and apoptosis [22]. Increasing evidence suggests that stem cell transplantation may decrease circulating inflammatory cytokines such as TNF-α, IL-6, and IL-1β in response to injury. Aggarwal et al. observed that immune cells co-cultured with MSCs may alter the cytokine secretion profile and finally lead to immunotolerance in vitro [23]. Among the candidates for positive regulators of cardiomyocyte differentiation, the TGF-β, IL-6, and chemokine families are the most widely investigated to date and have been demonstrated to be the key players in cardiomyocyte regeneration.

#### TGF-β and cardiomyocyte regeneration

The zebrafish heart shows a remarkable regenerative capacity compared to the mammalian heart. TGF-β is expressed during early cardiac development in zebrafish, which coordinates a wide spectrum of subsequent cellular steps that are required for efficient cardiac regeneration. Recently, it was reported that Smad3-dependent TGF-β signaling orchestrates beneficial effects on cardiomyocyte-based regeneration to achieve complete heart regeneration [24]. Downregulation of TGF-β/activin signaling caused a wide range of cellular phenotypes affecting heart function. In MI, TGF-β plays a pivotal role in cardiac repair by suppressing inflammation and promoting the myofibroblast phenotype and extracellular matrix deposition. Myofibroblast proliferation results in restoration of cardiac function after MI. Moreover, TGF-β simultaneously induced myogenesis and inhibited adipogenesis in a dose-dependent manner [25]. In MI, TGF-β was upregulated especially in the infarct border zone associated with Smad2, 3, and 4 expression and phosphorylation of Smad1 and 2 [26]. TGF-β1 can induce MSCs to differentiate into either chondrocytes or SMCs in vitro [27, 28]. TGF-β released from MSCs can promote angiogenesis by stimulating EC proliferation. However, cell − cell and cell − matrix interactions are necessary for such differentiation to occur, similar to development of these tissues in vivo [29]. TGF-β1 inhibits adipogenic differentiation of MSCs in monolayer culture. Recently, Rouhi et al. demonstrated that autologous serum enhances cardiomyocyte differentiation of rat bone marrow MSCs cells in the presence of TGF-β1 [30]. Taken together, the TGF-β1 signaling pathway may serve as a potential therapeutic target for the heart regeneration process.

#### IL-6 family of cytokines and cardiomyocyte regeneration

Accumulating data show that the IL-6 family also plays a key regulatory role in cardiomyocyte regeneration. The IL-6 family includes leukemia inhibitory factor (LIF), cardiotrophin-1 (CT-1), IL-6, oncostatin-M, and neutrotrophin-1/B-cell stimulating factor-3 (NNT-1/BSF-3) [31, 32]. IL-6 has numerous activities. On target cells, IL-6 binds to an 80 kDa IL-6 receptor (IL-6R) associated with a second protein, gp130, and initiates intracellular signaling [33]. IL-6 has multiple regulatory functions in the immune and nervous systems. Furthermore, IL-6 is also involved in liver regeneration and metabolic homeostasis [34]. Exogenous recombinant IL-6 influences the proliferation and differentiation of cultured myoblasts derived from human or murine muscle via the activation of the STAT3 signaling pathway [35]. Our group recently demonstrated that IL-6 expression and activation of the STAT3 signaling pathway in monocytes/macrophages are critical mediators of macrophage migration and myoblast proliferation during muscle regeneration [36].

CT-1 is another member of the IL-6 cytokine family. CT-1 knockdown did not affect infarct size in a murine model, suggesting that CT-1 does not play an essential role in MI-related injury. Upregulation of CT-1 in the infarcted myocardium modulates the fibrotic response through the inhibition of fibroblast proliferation [37]. However, CT-l activates the Janus kinase/signal transducers and activators of transcription (JAK/STAT), mitogen-activated protein (MAP) kinase, phosphatidylinositol (PI) 3 kinase, and nuclear factor κB (NF-κB) pathways to exert its hypertrophic and cytoprotective properties [38].

#### Chemokines and cardiac regeneration

Chemokines comprise a family of small, highly basic proteins with strikingly similar tertiary structures [39]. Chemokines are markedly upregulated in healing myocardial infarcts and play important roles in modulating infarct angiogenesis and fibrous tissue deposition. In the CXC chemokine subfamily, the SDF-1α/CXC-chemokine receptor type 4 (SDF-1α/CXCR4) axis is the best-understood signaling pathway for cardiogenesis, neovascularization, hematopoiesis, and neuronal development, as well as endothelial progenitor cell trafficking. SDF-1 is predominantly expressed in the infarct area after MI, which causes the migration of MSCs into the heart and may responsible for the improvement of cardiac function [40]. We previously demonstrated that endothelial nitric oxide synthase (eNOS) promotes the migration of MSCs toward the infracted heart through the upregulation of SDF-1 [41]. However, this SDF-1 − induced MSCs migration toward the MI area can nearly be abolished by a PI3K inhibitor and CXCR4 antagonist [40]. Overexpression of SDF-1 in MSCs promoted angiogenesis and improved cardiac function in a rat MI model [42].

### Cytokines, neovascularisation, and angiogenesis

MI induces inflammation in the infarcted zone and the accumulation of various factors that promote the angiogenesis process. Lai et al. demonstrated that MSC-derived microparticles increase myocyte viability and reduce adverse remodeling after myocardial hypoxia-induced injury [43]. These microparticles has been demonstrated to contain cytokines, chemokines, and microRNAs. Neovascularisation consists of two district processes, vasculogenesis and angiogenesis. Vasculogenesis is driven by bone marrow-derived circulating endothelial progenitor cells whereas angiogenesis is initiated by local ECs proliferating from existing vasculature. Neovascularisation is stringently regulated by a variety of cytokines, including the VEGF, FGF, and Ang families [44]. Here, we summarize the major cytokines that play pivotal roles in neovascularisation and angiogenesis.

#### VEGF

VEGF plays crucial roles in EC proliferation and recruitment, embryonic development, and ischemic tissue damage. VEGF acts through the VEGF receptor (VEGFR) 1 or VEGFR 2 protein tyrosine kinases. Hypoxia-inducible factor (HIF)-1 is the key regulator of VEGF expression [45, 46]. Five human VEGF isoforms (A, B, C, D, and placental growth factor [PlGF]) have been produced by differential splicing of VEGF mRNA. VEFG-A is primarily involved in vascular growth, lymphatic development, and vascular malformation [47]. Thus VEGF-A has been intensively investigated regarding its role in tumorigenesis and potential for cancer therapy. While VEGF-C and VEGF-D regulate the lymphangiogenesis [48]. VEGF appears to be a critical regulator of EC proliferation [49]. Pretreatment of cardiac scars with gene transfer of VEGF in addition to the Gata4, Mef2c, and Tbx5 (GMT) enhanced the transdifferentiation of rat fibroblasts into (induced) cardiomyocytes [50]. The addition of VEGF into the transfection cocktail resulted in a 4-fold improvement in the cardiac ejection fraction [50]. Recently, Ye et al. demonstrated that VEGF is required for effective cardiomyocyte differentiation of human iPS [51]. Moreover, VEGF also mediates the phosphorylation of eNOS, which is pivotal in the regulation of angioblast and embryonic EC proliferation [52]. Taken together, VEGF is a key regulator of angiogenesis after cardiac ischemia and warrants further investigation as a therapeutic target for hypoxia-induced tissue damage in subsequent clinical trials.

#### FGF

The FGF protein family exhibits a wide variety of effects. Among these proteins, acidic FGF (FGF-1) and basic FGF (FGF-2) are the most critical stimulators of EC proliferation and angiogenesis promotion in cancer and cardiac hypoxia [53]. FGF is upregulated early after cardiac ischemic injury, which suggests that it may be involved in the regulation of the early inflammatory reaction after MI. Although FGF and VEGF activate different genes and seem to stimulate different vessel types, these actions are highly correlated [54]. It has been demonstrated that FGF’s angiogenic effects are VEGF dependent, while VEGF-induced tubulogenesis requires FGF signaling [55]. FGF has been shown to induce neovascularization in a rat vascular pedicle model [56]. Endothelial progenitor cells express FGF receptors and it has been demonstrated that FGF regulates the proliferation and differentiation of endothelial progenitor cell-like MSCs through the ERK1/2 signaling pathway [57]. In a murine diabetes model, the improvement of neovascularization corresponded to the high local expression of FGF and VEGF, suggesting that these cytokines play key roles in post-ischemic angiogenesis.

Recently, it was also revealed that MSCs overexpressing granulocyte chemotactic protein (GCP)-2 improved cardiac function through enhanced angiogenic properties in a myocardial infarction model [58]. Taken together, the angiogenic cytokines secreted by MSCs represent promising therapeutic targets for the treatment of MI patients.

### Cytokines and other cardioprotective effects

The cytokines secreted by MSCs also play important roles in cardiomyocyte apoptosis, cardiac contractility, cardiac remodeling, and inflammation.

#### Anti-apoptotic effects

Mirotsou et al. reported that secreted frizzled-related protein 2 (Sfrp2) promotes myocardial repair by increasing cellular catenin and upregulating expression of anti-apoptotic genes Birc1b and Bcl2 [59]. Wang et al. have demonstrated that Hsp20-engineered MSCs are resistant to oxidative stress due to enhanced activation of Akt and increased secretion of VEGF, FGF-2, and IGF-1 [60]. MSCs pretreated with a combination of growth factors, including FGF-2, IGF-1, and BMP-2, cause reduced cardiomyocyte apoptosis under hypoxic conditions and enhance the phosphorylation of Akt and cyclic adenosine monophosphate (cAMP), resulting in better gap junctions and decreased infarct size [61].

#### Anti-arrhythmic effects

The anti-arrhythmic potential of MSCs remains controversial. Chang et al. revealed the proarrhythmic potential of MSC transplantation in an in vitro co-culture system [62]. Such proarrhythmic effects may be related to the heterogeneity of MSCs, immaturity of transdifferentiated MSCs, and contractile dyskinesia between MSCs and in situ cardiomyocytes. However, Ly et al. contend that stem cell therapy promotes important anti-arrhythmic effects if properly applied [63]. Thus, studies regarding the effects of MSC-related cytokines on arrhythmia need further assessment. The anti-arrhythmic effects of MSCs may serve more important clinical applications than anti-arrhythmic drugs.

#### Anti-inflammatory effects

After infarct damage, several innate immune pathways are activated in the infarcted myocardium. Inflammatory factors such as TGF-β, IL-1β, IL-6, TNF-α, VEGF, and others are consistently found in the infarct area. Aggarwal et al. observed that MSC trafficking into the infarct area decreases the secretion of TNF-α and interferon-γ, while increasing IL-4 and IL-10 production [23]. MSC transplantation also attenuates the activity of NF-κB, inhibits the protein production of TNF-α and IL-6, and increases the expression of IL-10 in infarcted myocardium [64]. MSCs also release heme oxygenase-1 (HO-1) and eNOS, important anti-oxidative stress factors, resulting in the protection of cardiomyocytes, improvement of neovascularization, and improvement in cardiac function during the early stage after MI [65]. The mechanisms by which MSCs modulate the immune system to reduce the inflammatory response warrant further investigation.

## Conclusions

Myocardial regeneration has been intensively studied in the last decade given the promising results of the stem cell therapy in MI. Among the mechanisms underlying the cardioprotective effects of stem cells, the paracrine cytokines released by stem cells are the most important factors. This review summarized the major cytokines involved in cardiomyocyte differentiation, angiogenesis, and neovascularization, as well as anti-apoptotic and anti-arrhythmic processes. Increased understanding of the cardioprotective mechanisms of MSCs and iPS may enable the discovery of more beneficial cytokines for heart regeneration. The full understanding of the related cytokine signaling pathways and their complex biological effects will be essential for future clinical applications.
